# The First Case of Ceftazidime-Induced Antibiomania: A Case Report and Literature Review

**DOI:** 10.7759/cureus.32904

**Published:** 2022-12-24

**Authors:** Sujith K Palleti, Anuradha Wadhwa, Abdul H Rasheed

**Affiliations:** 1 Nephrology, Edward Hines Jr. Veterans Administration Hospital, Hines, USA; 2 Nephrology, Loyola University Medical Center, Maywood, USA

**Keywords:** antibiotic induced psychosis, quinolone induced mania, macrolide induced mania, cephalosporin induced mania, clarithromycin induced mania, mania psychosis peritoneal dialysis, antibiotic psychosis, antibiotic induced mania, ceftazidime toxicity, antibiomania

## Abstract

Antibiomania is a rare type of secondary mania caused by antibiotics. Here, we describe the first case report of antibiomania in a peritoneal dialysis patient caused by ceftazidime, a cephalosporin antibiotic. Manic symptoms were noted in the patient described here a few days after starting the antibiotic and worsened over the course of a few weeks of antibiotic treatment. There is a close temporal relationship between the start of antibiotics and the onset of manic symptoms in this case. Antibiotics from various groups have been implicated as the causative agents in a few case reports of antibiomania. Several hypotheses have been postulated regarding the underlying mechanism of antibiomania. The authors of this article highlight the importance of recognizing the psychiatric side effects of antibiotics early and formulating effective management options to prevent detrimental effects on a patient's clinical condition.

## Introduction

Antibiomania is a term first introduced in 2002 [1]. It is a type of secondary mania induced by antibiotics and a relatively rare condition given the widespread use of antibiotics and the few reported cases. Mania can occur with different somatic conditions or the use of medications in patients with or without a history of affective disorder. In the fifth edition of the Diagnostic and Statistical Manual of Mental Disorders (DSM-5), these cases are best classified as "substance/medication-induced bipolar and related disorders." The worldwide use of antibiotics has increased by 36% between 2000 and 2010, with broad-spectrum penicillin being the most used antibiotic in 2010, followed by cephalosporins, macrolides, and quinolones. To date, macrolides (such as clarithromycin), quinolones (such as ciprofloxacin and ofloxacin), and antitubercular drugs are the most common causative agents of antibiomania reported [2]. A few cases are associated with several other antibiotics.

This article was previously presented as a conference abstract at the ASN Kidney Week 2022 on November 4, 2022.

## Case presentation

A 55-year-old male with a past medical history of end-stage kidney disease (ESKD) due to diabetes and hypertension on peritoneal dialysis (PD) and a history of stable depression without medications for over a year with no other psychiatric illness presented to the PD clinic with abdominal pain, and the workup revealed PD-related peritonitis (Table 1). He was started on intraperitoneal (IP) ceftazidime for gram-negative rods as culture showed Capnocytophaga, and his peritonitis improved with a reduction in white blood cell count. On day 12 of treatment, changes in his behavior were noted, including agitation, anxiety, and tearfulness. Psychiatry was consulted and recommended starting sertraline for depression. The patient was sent home to finish the three weeks of ceftazidime.

**Table 1:** Peritoneal fluid analysis during the first and second peritonitis episodes

	Reference range	February 18, 2021	February 21, 2021	April 9, 2021	April 11, 2021
Color	Yellow	Light yellow	Light yellow	Colorless	Light yellow
Appearance	Clear	Cloudy	Clear	Clear	Cloudy
Nucleated cells	< 300/micL	2694	630	214	7251
Red blood cells	< 2000/micL	< 2000	< 2000	< 2000	< 2000
Neutrophils	< 25%	78	52	49	95
Lymphocytes	20 – 80%	3	4	14	1
Monocytes/Macrophages	20 – 80%	15	43	35	3
Eosinophils	< 25%	1	1	1	1
Mesothelial cells	20 – 80%	3	1	1	1
Culture sensitivity	No growth	Capnocytophaga	No growth	E. cloacae complex	E. cloacae complex

He was readmitted four weeks later due to disruptive behavior with delusions, agitation, elevated mood, reduced sleeping hours, and increased energy. Psychiatry diagnosed him with mania due to ceftazidime and started him on divalproex sodium. PD fluid showed a recurrence of peritonitis with 7251 WBCs, 95% neutrophils, and Enterobacter cloacae on culture. He was treated with cefepime, but due to abdominal pain and increasing WBC, the PD catheter was removed. He transitioned to hemodialysis and completed two weeks of IV cefepime. His mental status was monitored while on divalproex sodium. After a year of therapy with antipsychotics, his mood remained stable, and he has switched back to PD.

## Discussion

The patient presented here had developed symptoms of mania after the administration of the antibiotic IP ceftazidime for PD-related peritonitis. Peritonitis is a common complication in peritoneal dialysis patients that is associated with significant morbidity [3]. His symptoms are characterized by disruptive behavior with delusions, irritability, and agitation. In antibiomania, a close temporal relationship is usually present between the initiation of the antibiotic and the onset of manic symptoms, as witnessed in this case. The underlying psychiatric condition may or may not always be present. Remission of manic symptoms with discontinuation of the causative agent has only been reported in one-third of cases; hence, treatment with antipsychotics may be required.

Several hypotheses have been postulated regarding the underlying mechanism of antibiomania [1]. Gamma-aminobutyric acid (GABA) neurotransmission is inhibited by antagonizing its receptor, which is involved in the manifestation of CNS toxicity with beta-lactam antibiotics such as ceftazidime [4]. GABA neurotransmission, the predominant inhibitory neurotransmitter in the CNS, acts to reduce neuronal excitability [5]. Among penicillins, piperacillin and tazobactam appear to be most associated with neurotoxic events, with several reported cases. Hoigne's syndrome is an acute psychotic, toxic, nonallergic reaction most commonly associated with procaine penicillin [6]. Patients with advanced renal failure are particularly at increased risk. Quinolones can cross the blood-brain barrier (BBB) [7] and competitively inhibit the binding of GABA to their receptor sites [8].

Isoniazid, an antitubercular drug, also crosses the BBB and decreases GABA concentration by inhibiting glutamic acid decarboxylase (GAD), an enzyme that catalyzes the decarboxylation of glutamate to GABA and CO2 [9]. Clarithromycin, a macrolide antibiotic, antagonizes postsynaptic GABA-A receptors in neurons and increases neuron excitability [10-14]. Several case reports of secondary mania induced by clarithromycin have been published in the past [15-17].

## Conclusions

As far as we are aware, this is the first case report of antibiomania in a peritoneal dialysis patient caused by the antibiotic ceftazidime. This case report highlights the importance of recognizing the psychiatric side effects of antibiotics early and formulating effective management options to prevent detrimental effects on a patient's clinical condition. Antibiomania should be strongly considered as a differential diagnosis in patients presenting with manic symptoms after the initiation of ceftazidime.

## References

[1] Abouesh A, Stone C, Hobbs WR (2002). Antimicrobial-induced mania (antibiomania): a review of spontaneous reports. J Clin Psychopharmacol.

[2] Lambrichts S, Van Oudenhove L, Sienaert P (2017). Antibiotics and mania: a systematic review. J Affect Disord.

[3] Palleti SK, Bavi SR, Fitzpatrick M, Wadhwa A (2022). First case report of Sphingobium lactosutens as a human pathogen causing peritoneal dialysis-related peritonitis. Cureus.

[4] Mattappalil A, Mergenhagen KA (2014). Neurotoxicity with antimicrobials in the elderly: a review. Clin Ther.

[5] Bichler EK, Elder CC, García PS (2017). Clarithromycin increases neuronal excitability in CA3 pyramidal neurons through a reduction in GABAergic signaling. J Neurophysiol.

[6] Guarneri C, Tchernev G, Wollina U, Lotti T (2017). Hoigne syndrome caused by intralesional meglumine antimoniate. Open Access Maced J Med Sci.

[7] HeβLinger B, Hellwig B, Sester U, Walden J, Berger M (1996). An acute psychotic disorder caused by pefloxacin: a case report. Prog Neuropsychopharmacol Biol Psychiatry.

[8] Tsuji A, Sato H, Kume Y, Tamai I, Okezaki E, Nagata O, Kato H (1988). Inhibitory effects of quinolone antibacterial agents on gamma-aminobutyric acid binding to receptor sites in rat brain membranes. Antimicrob Agents Chemother.

[9] Carta M, Murru L, Barabino E, Talani G, Sanna E, Biggio G (2008). Isoniazid-induced reduction in GABAergic neurotransmission alters the function of the cerebellar cortical circuit. Neuroscience.

[10] Ortíz-Domínguez A, Berlanga C, Gutiérrez-Mora D (2004). A case of clarithromycin-induced manic episode (antibiomania). Int J Neuropsychopharmacol.

[11] Przybylo HJ, Przybylo JH, Todd Davis A, Coté CJ (2005). Acute psychosis after anesthesia: the case for antibiomania. Paediatr Anaesth.

[12] Pilla R, Palleti SK, Rayana R, Reddy SR, Abdul Razzack A, Kalla S (2020). Glycated haemoglobin (HbA1c) variations in nondiabetics with nutritional anemia. Cureus.

[13] Ozsoylar G, Sayin A, Bolay H (2007). Clarithromycin monotherapy-induced delirium. J Antimicrob Chemother.

[14] Brooks JO 3rd, Hoblyn JC (2005). Secondary mania in older adults. Am J Psychiatry.

[15] Abouesh A, Hobbs WR (1998). Clarithromycin-induced mania. Am J Psychiatry.

[16] Cone LA, Sneider RA, Nazemi R, Dietrich EJ (1996). Mania due to clarithromycin therapy in a patient who was not infected with human immunodeficiency virus. Clin Infect Dis.

[17] Nightingale SD, Koster FT, Mertz GJ, Loss SD (1995). Clarithromycin-induced mania in two patients with AIDS. Clin Infect Dis.

